# A case of angiosarcomas which occurred in an adrenal gland and spleen synchronously

**DOI:** 10.1007/s13691-018-0337-y

**Published:** 2018-07-06

**Authors:** Shuhei Ishii, So Omori, Noriyuki Uesugi, Takashi Tsuyukubo, Ayato Ito, Daichi Kikuchi, Mitsutaka Onoda, Ryo Takata, Tamotsu Sugai, Wataru Obara

**Affiliations:** 1 Department of Urology, Iwate Medical University School of Medicine, 19-1 Uchimaru, Morioka, Iwate 020-8505 Japan; 2 Department of Molecular Diagnostic Pathology, Iwate Medical University School of Medicine, 19-1 Uchimaru, Morioka, Iwate 020-8505 Japan

**Keywords:** Angiosarcoma, Adrenal gland, Spleen, Simultaneous occurrence

## Abstract

We report a case of a 56-year-old woman who simultaneously presented adrenal and spleen tumors. Computed tomography imaging revealed a 7-cm enhancing adrenal and 2-cm solitary spleen masses. The patient simultaneously underwent left adrenalectomy and splenectomy. The pathological findings revealed the presence of synchronous adrenal and spleen angiosarcomas. Remarkably, she is disease-free since postoperative 18 months.

## Introduction

Angiosarcoma, a malignant endothelial-derived soft-tissue sarcoma, develops from blood or lymph vessels, with the most common locations being the head, neck, breast, and extremities [1]. Although surgical resection is the standard treatment for angiosarcoma, the 5-year survival rate remains at a dismal 30% [2]. Adrenal and spleen angiosarcomas are sporadic diseases, and adjuvant therapy such as chemotherapy or radiotherapy has been employed to address its aggressive biology. To the best of our knowledge, this is the first report of a case involving angiosarcomas with a simultaneous occurrence in the spleen and left adrenal gland.

## Case report

**Fig. 1:**
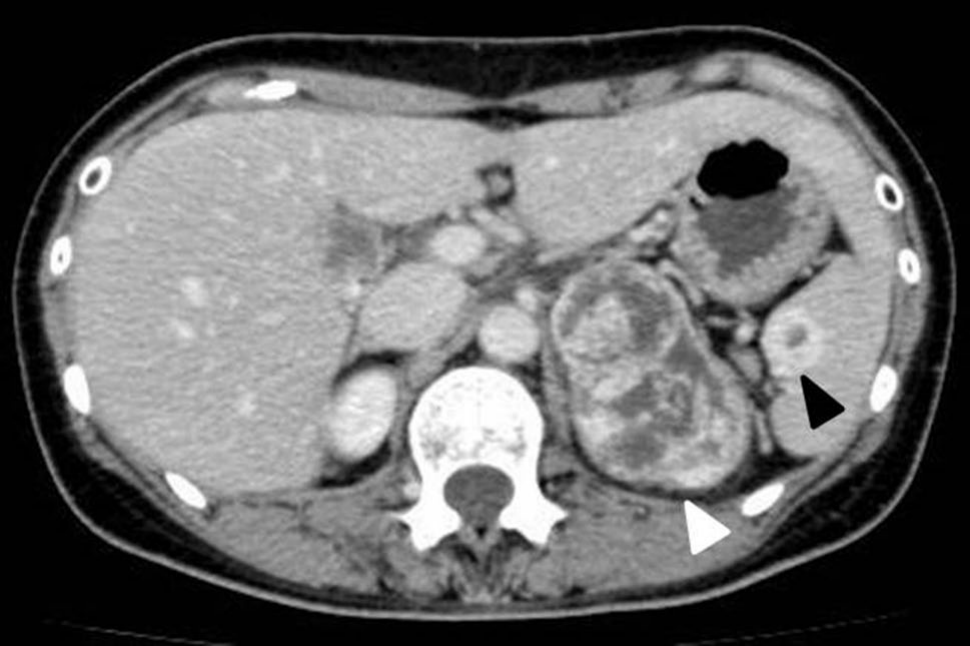
Computed tomography examination showing a heterogeneously enhancing left adrenal tumor with several septa (white allow) and an enhanced spleen mass (black allow)

**Fig. 2:**
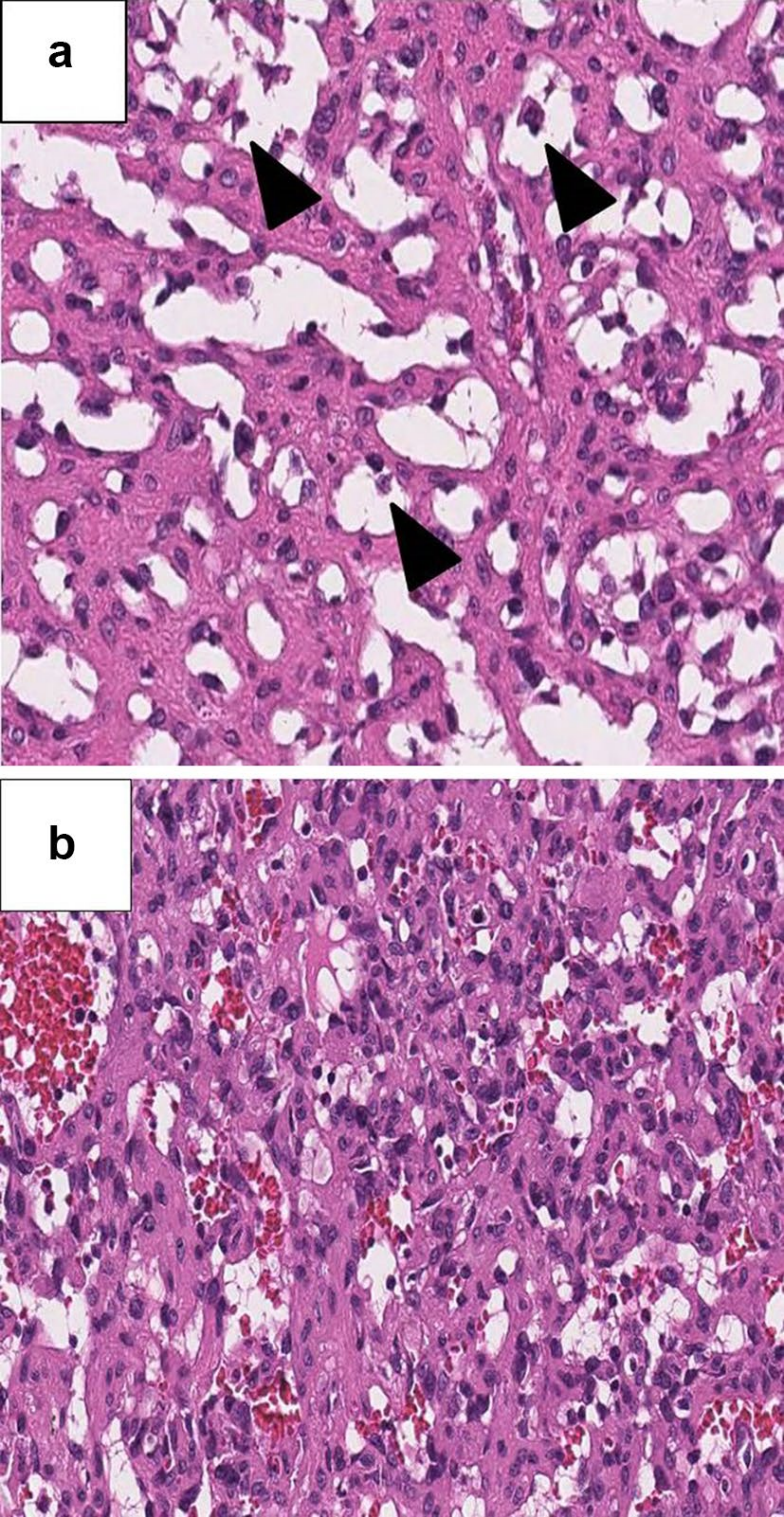
In hematoxylin and eosin staining, adrenal tumor demonstrated epithelioid cells with eosinophilic cytoplasm. Some tumor cells (black arrows) were observed within the blood vessels (**a**). The spleen tumor also demonstrated round epithelioid cells with eosinophilic cytoplasm and large hyperchromatic nuclei with prominent nucleoli (**b**) (magnification, ×300)

A 56-year-old woman visited our hospital presenting a left adrenal tumor; during examination, a spleen nodule was incidentally diagnosed. Computed tomography revealed a 7.4-cm-long enhanced left adrenal tumor and 1.8-cm-long enhanced nodule within the spleen (Fig. 1). Lymph node and other organ metastases were absent, and adrenal endocrine examination findings (serum cortisol, renin, aldosterone, testosterone, metanephrine, DHEA-S, and 17-OH progesterone) were within the normal ranges. Open left adrenalectomy and splenectomy were simultaneously performed, and the adrenal tumor and spleen were separated from each other; notably, the perioperative findings demonstrated no direct invasion. As per gross examination, the adrenal tumor was reddish-brown and covered with multiple white capsula fibrosa. The cut surface of the spleen nodular tumor was also reddish-brown. The pathological findings of the adrenal tumor demonstrated the presence of epithelioid cells with eosinophilic cytoplasm; some tumor cells were found within the blood vessels (Fig. 2a). The spleen tumor also demonstrated round epithelioid cells with eosinophilic cytoplasm and large hyperchromatic nuclei with prominent nucleoli (Fig. 2b). Immunohistochemical analysis revealed that the adrenal and spleen tumors were positive for CD31, CD34, and factor VIII. The final pathological diagnosis was angiosarcomas with a simultaneous occurrence in the spleen and left adrenal gland. The patient was alive and in remission at postoperative 18 months.

## Discussion

Angiosarcoma, a rare disease developing from the endothelium of blood or lymph vessels, constitutes 1–2% of all sarcomas [3]. This tumor can be incidentally diagnosed during imaging and often appears in an advanced disease state. The histological features of angiosarcoma show various morphologies, as abnormal endothelial cells are the hallmark of angiosarcoma, possessing rounded, polygonal, or fusiform patterns and predominantly exhibiting an epithelioid appearance. With gradual progression, tumors develop into a highly complex architecture without a clear vascular construction. Most immunohistochemical findings were positive for endothelial markers such as von Willebrand factor, CD31, CD34, and factor VIII [1]. In our case, these molecules were concurrently positive in the adrenal and spleen tumors.

Primary adrenal angiosarcoma is extremely rare and was first reported by Kareti et al. in 1988 [4]. Since then, an additional 40 cases have been reported in the literature with a median age at presentation of 60 (range, 34–85) years [5]. The most common symptom associated with adrenal angiosarcoma is an abdominal mass-accompanied pain. Notably, none of the reported cases included hyperfunctioning tumors. Adrenal angiosarcoma often includes high-grade tumors possessing a potential to infiltrate and metastasize [2]. Adrenalectomy serves diagnostic and therapeutic purposes. Reportedly, the median survival for patients with localized disease is only 7 months [1]. Previously reported multimodal treatment approaches included doxorubicin-based chemotherapy and adjuvant radiation therapy [5]. Although we recommended adjuvant therapy for our patient, she refused it. Primary spleen angiosarcoma is also aggressively malignant with a rare incidence of 0.14–0.23 cases per million. Although there is no specific chemotherapeutic regimen, splenectomy is the preferred treatment for localized disease [6]. Similar to adrenal angiosarcoma, the prognosis in spleen angiosarcoma is extremely poor. To the best of our knowledge, only 200 cases are reported in the literature to date [6] with a survival time of only approximately 5 months from diagnosis [7]. Primary origin in the spleen is defined as no metastasis during presentation as determined by sophisticated radiological procedures and surgical staging or massive tumor burden in the spleen. There can often be multiple target lesions of metastasis, including the lungs, liver, lymph nodes, bones, and adrenal glands [8].

In our case, differential diagnosis was adrenal carcinoma and spleen metastasis, because the adrenal tumor size was > 4 cm. According to the report from Alfonso et al., the tumor size more than 4 cm indicates malignancies [9], although the determination of malignant potential of the adrenal and spleen lesions was not performed. The adrenal tumor was larger than the spleen tumor. Pathologically, some tumor cells with a low nucleus-to-cytoplasm ratio and low nuclear grade were observed in the adrenal angiosarcoma, suggesting that the adrenal gland was the primary lesion. Hematogenous spread is considered the most probable route of spread, resulting in spleen metastasis [10]. Given the rarity of the disease, there is no consensus on the best treatment modality for adrenal and spleen angiosarcomas [5]; however, surgery is still the main stay of treatment [11] and it was considered that we might not be reluctant to perform surgery if patients consent to undergo surgery.

To the best of our knowledge, this is the first report of angiosarcomas simultaneously occurring in the left adrenal gland and spleen. The diagnosis was readily established with appropriate H&E and immunochemical panels. The patient underwent surgery and survived for 18 months. Hence, we believe that surgery should be considered when patients are tolerant undergoing surgery.
